# Enhanced method for High Spatial Resolution surface imaging and analysis of fungal spores using Scanning Electron Microscopy

**DOI:** 10.1038/s41598-018-34629-8

**Published:** 2018-11-02

**Authors:** Gopal Venkatesh Babu, Palani Perumal, Sakthivel Muthu, Sridhar Pichai, Karthik Sankar Narayan, Sathuvan Malairaj

**Affiliations:** Centre for Advanced Studies in Botany, University of Madras, Chennai, 600025 India

## Abstract

Efficient, fast and new micro-analytical methods for characterization of ultrastructures of fungal spores with electron microscopy are very much required and essential. SEM analysis of biological materials, especially fungi, requires optimal preparation of the specimen and often requires the usage of dried samples which demands a challenging sample preparation. In the present investigation, we described a fast and improved method for the preparation of fungal specimen for scanning electron microscopy (SEM). The fungus, *Curvularia lunata* was grown on the surface of sterile Whatman No.1 filter paper which was overlaid on Potato Dextrose Agar (PDA) medium, gold coated immediately after removal from the growth medium and subjected to imaging. Generally, SEM imaging is done with samples that were fixed with chemical fixatives, dehydrated and gold coated specimens, but here we describe an easy and more efficient sample preparation for SEM which enabled enhanced image quality and precision visualization of fungal cells, especially the spores. The developed method has enabled the analysis of even the robust samples like fungal spores that to eliminating special temperature requirement. The ultimate goal was to develop an improved protocol/method applied to analysis of fungal spores with greater coverage about fungal specimen preparation. This method permits the use of rapid sample preparation and will allow us to imaging of individual spore or conidia structures in the context of fungal cell architecture which clarifies our understanding in fungal taxonomy/biology.

## Introduction

Scanning Electron Microscopy (SEM) is a prototypical and yet a powerful technique for studying the topological characteristics of biological samples with high resolution and it has revolutionized the imaging analysis of not only samples of biological origin but other samples as well. Scanning Electron Microscopy offers several advantages which includes bi-dimensional aspect of the image with high depth of field, large increase in magnitude from 10 to 1,000,000 times, digitalization, rapid image processing and acquisition, ease of preparation of sample and operation accessible cost^1,2^. The quality and resolution of images in a SEM directly depends on three vital factors, fixation, post fixation and dehydration. Scanning Electron Microscopic analysis is generally performed for the identification of microorganisms and especially the taxonomical identification of fungal species which is done mainly based on the morphological characteristics of the spores produced by them. The fungal cells must be perpetuated by fixation and subsequent dehydration for examining under the electron microscope since the processes like subjecting the samples under ion sputter and imaging are usually performed under high pressure vacuum and most of the biological specimens cannot combat pressure generated by the high vacuum system which leads to the distortion of sample^3^. In order to examine the natural/ true structure of the biological sample, some microscopes are intended to image cryofixed hydrated samples and in recent time ESEM (Environmental SEM) microscopes have been developed which has the potential to image the sample in their natural state^4^. But, these microscopes are specially designed equipments which may not be feasible in many laboratories. Therefore, preparation of biological sample by dehydration is yet a prime requirement for observation under conventional microscopes. For the specimens that impel numerous techniques other than just employing air-drying have been developed to remove the moisture content from the sample, with an intent to have minimal shrinkage and maximal structural preservation in their original form. These techniques comprise of cryofixation followed by freeze-drying, critical point drying and discrete types of chemical fixation treatments prior to dehydration of samples^5^. However, a suitable method offers a comparatively less than ideal/native preservation for a few parts of the fungal cells and may be contrary. The contradictory is mainly due to the heterogeneity in its structure, anatomy and composition of fungal cell. However, the existing protocols may have to be refined and adjusted according to the nature of the biological samples and also depends on the individual handling skills and availability of equipment across different laboratory. So an innovative and modified technique are constantly being examined and refined for the design of preservation strategies of fungal spore sample optimum for SEM imaging, which includes hydrated, dehydrated/dried, chemical fixation, frozen hydrated, freeze drying, simple air drying and critical point drying of the biological sample^5–7^. In the present study, a rapid, easy to follow and time saving method has been developed for the preparation of fungal sample for image analysis with SEM and the results obtained have been in contrast with those from the preceding studies, where a similar techniques for imaging fungal sample with electron microscopy have been used. The developed method involves growing the fungal sample on a small strip of Whatman No.l filter paper which was overlaid on potato dextrose agar (PDA) medium and drying under room temperature proved highly effective for visualisation of fungal spore in their native state without compromising the structural characteristics of the spores in a scanning electron microscope (SEM). For any spatially resolved or spatially selective method that does not analyze samples in their natural, hydrated state, the influence of drying on the sample structure has to be contemplated for imaging studies. In this regard, imaging methods such as scanning electron microscopy (SEM), transmission electron microscopy (TEM), helium ion microscopy (HIM), spectromicroscopy such as scanning transmission X ray microscopy (STXM) and energy-dispersive X-ray spectroscopy (EDX) mapping spectroscopy such as EDX spot measurements have aided significantly to the mycological research. There have been a number of technical advancements that would likely have application to fungi in the very near future, where the theme of integrated instrumentation is predominant^8,9^.

In this study, we present a pipeline to generate reproducible high spatial fungal spore imaging sample preparation by imaging with Scanning Electron Microscopy (SEM). This pipeline is reliable to implement and preserves fungal spore and cell morphology without disturbing its native structure. Here, we illustrate five successful approaches for this strategy, ranging from the different sample preparation and fixative to its subsequent SEM imaging.

## Results

### Preservation of fungal spore structures

The external structure of fungal spore to be examined was well preserved by all approaches. As molecular taxonomy moves toward studies of more complex systems, especially analysing the spores, the focus of interest has moved towards developing robust fungal samples preparation. Although there are various methods for imaging with structural integrity of the sample, the success of the experiment depends critically on the quality of the (specimen) (fungal spore) preservation. The fungal spores grown on upper surface of Whatman No.1 filter paper were used for all the approaches. Promising results have been obtained with very difficult and fragile spore samples.

### Visualization of sample in its native state without any treatment

**Figure 1:**
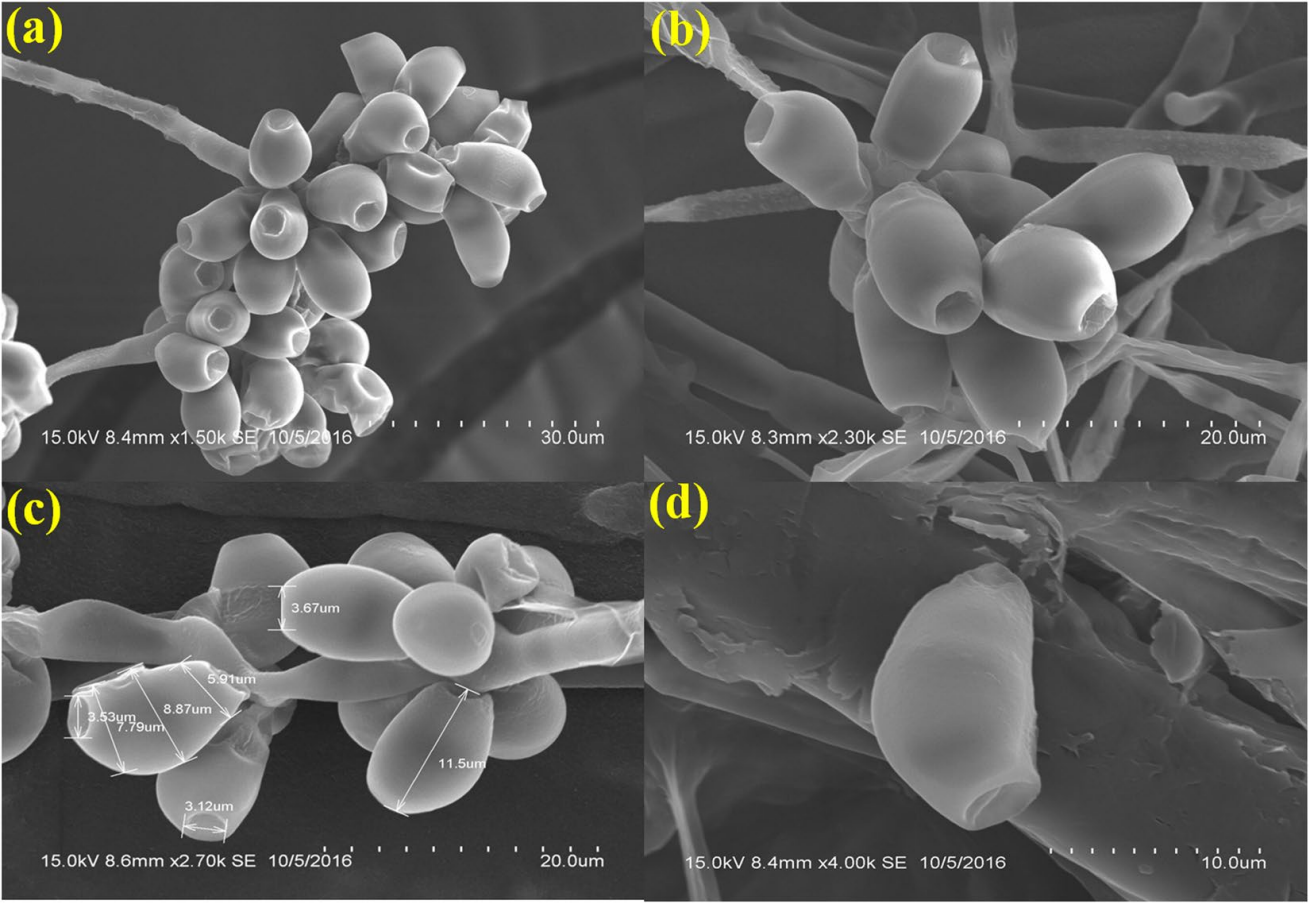
SEM analysis of *Curvularia lunata* spores processed by growing the fungus on Whatman No.1 filter paper strips overlaid with PDA medium with simple air drying (Native state). (**a**) Condiospores showing smooth wall and uniform density (scale bar = 30 μm). (**b**) A bunch of conidia without any perturbation (scale bar = 20 μm). (**c**) Spore dimension analysis in their native preserved form (scale bar = 20 μm). (**d**) Morphological characteristics of a single spore (scale bar = 10 μm).

The fungus grown on Whatman No.1 filter paper and then simple air drying gave an acceptable and superior preservation of fungal spore structures, shape, thickness and more consistently the size of the spores were comparatively better preserved than other methods tested. Excellent high resolution and structural artefact- free upscaled images with sharp and natural structure of the fungal spore were obtained with nominal or trivial surface contortion. We observed either no substantial shrinkage or severely distorted spore architecture. Moreover, this method effectively preserved its actual native structure without any major distortion. We also examined the variations in the fungal spores in terms of shape, size and structure of the spores which were subjected to different treatments. These variations were precisely compared with native spore structure of the fungus by analyzing the spore dimension (μm). In the Native/Ideal state, the spores were clear, composed of branched, septate, smooth walled, clavate and almost always slightly curved at the third cell from the base which is larger than the others and often paler near the apex. Moreover, high resolution and brightness of the fungal spore was also achieved. The spores were 11.5 μm long, 5.9 μm thick in the middle (the broadest part) and 3.53 μm inward groove at the apex (Fig. 1).

### Visualization of sample after chemical fixation

**Figure 2:**
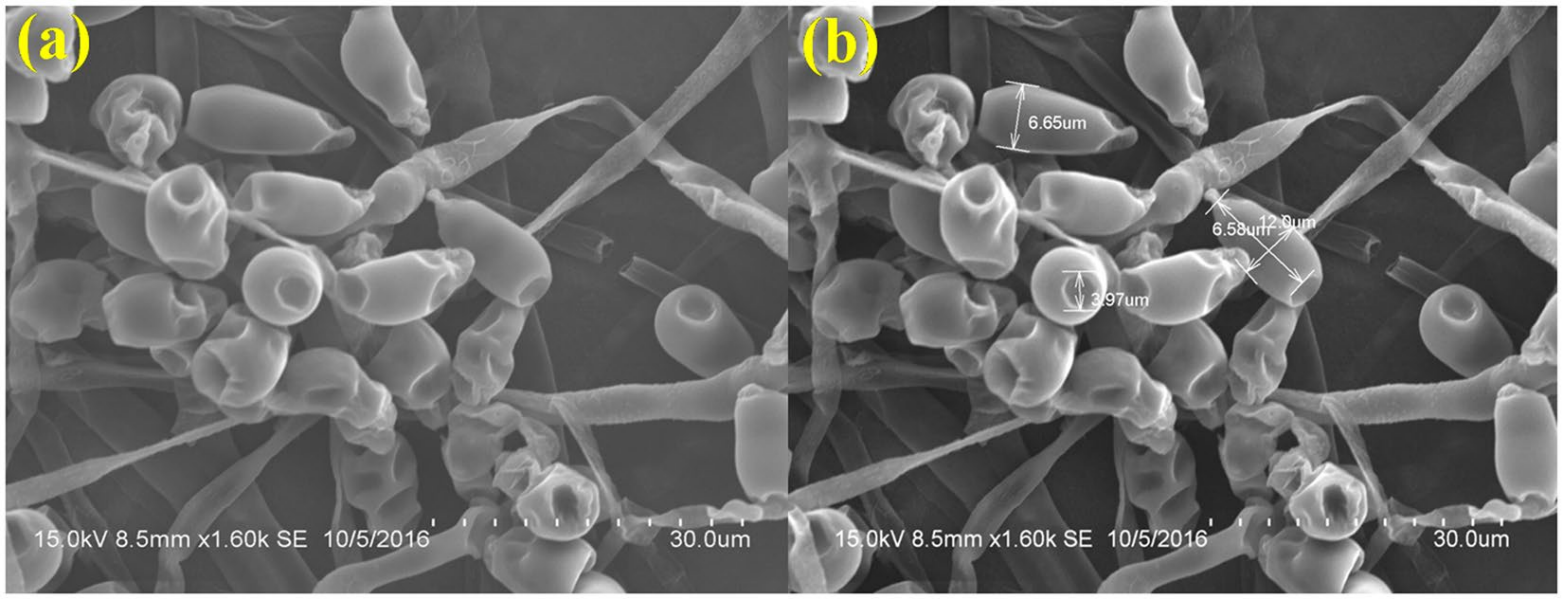
SEM analysis of *Curvularia lunata* spores processed by chemical fixation with glutaraldehyde followed by dehydration in ethanol and simple air-drying. (**a**) Fungus spore severely distorted and extensively shrinked by glutaraldehyde fixation (Scale bar = 30 μm). (**b**) The dimension analysis of damaged spores (Scale bar = 30 μm).

During chemical fixation, most of the spores were dehydrated and some spore were partially preserved in terms of size which is quite evident from the picture shown in Fig. 2 and most of the spores were zapped in the middle septate. The variation in spore dimensions during Glutaraldehyde fixation - The spore were 12.0 μm long, 6.58 μm thick in the middle (the broadest part) and 3.97 μm inward groove at the apex (Fig. 2).

### Visualization of sample involving chemical fixation and post fixation

**Figure 3:**
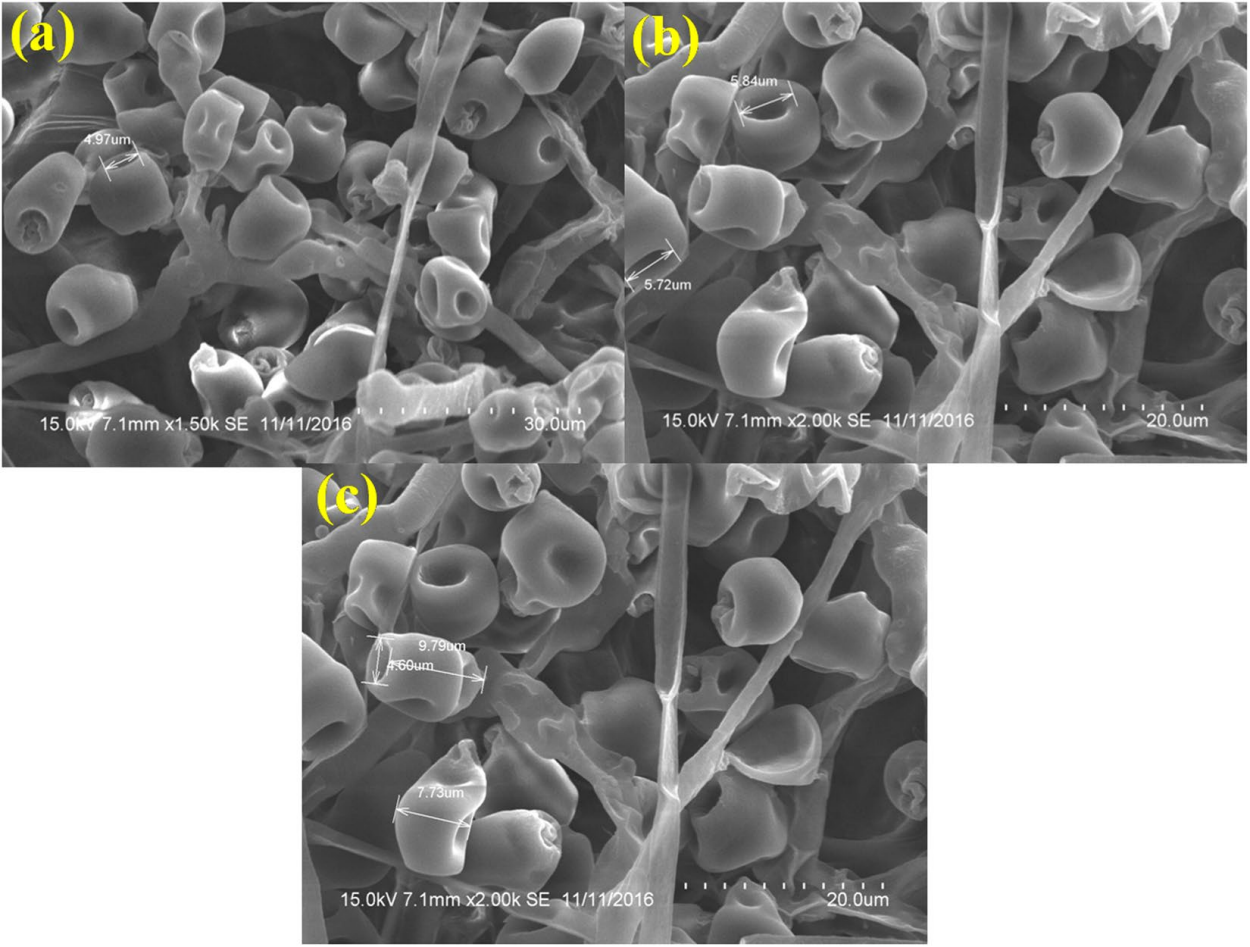
SEM analysis of *Curvularia lunata* spores processed by fixation with osmium tetroxide followed by dehydration in ethanol and simple air-drying. (**a**) Fungal spores were severely zapped out by the treatment of osmium tetroxide (Scale bar = 30 μm). (**b**) Spore deformed in structures (Scale bar = 20 μm). (**c**) Spore dimension analysis after treatment (Scale bar = 20 μm).

The samples postfixed with osmium tetroxide (O_2_O_4_) resulted in erroneous/unacceptable specimen preservation and have caused substantial alternation in the spore structure and heavy distortion in the outer layer which led to the deformation of the spores. Shrinkage on spore surface around the septum of the spore was also observed. This treatment totally collapsed and distorted the micro structure of the spores. The variation in spore dimensions were 9.73 μm long, 7.73 μm thick in the middle (the broadest part) and 4.97 μm inward groove at the apex (Fig. 3).

### Visualization of sample involving flash freezing

**Figure 4:**
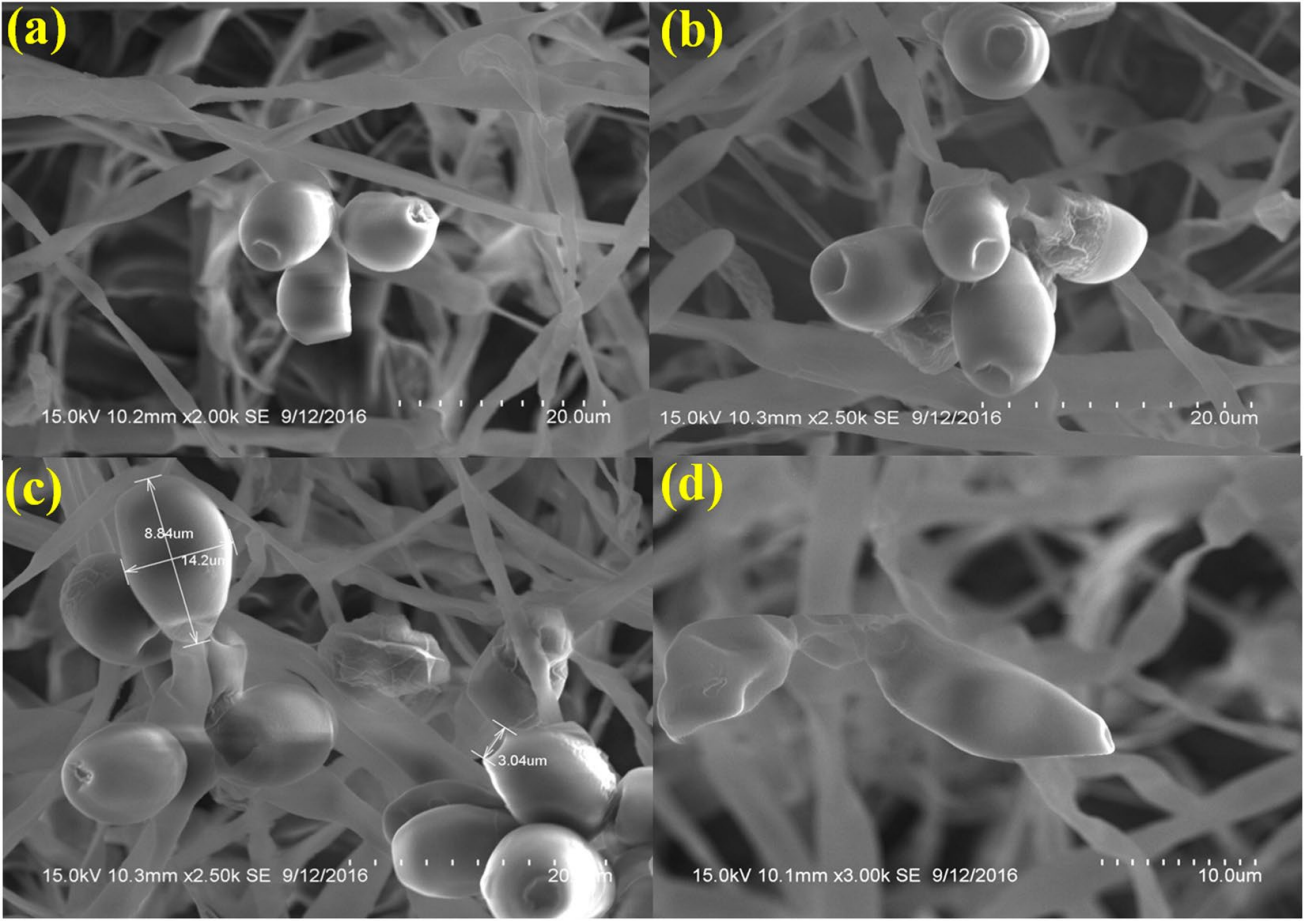
SEM analysis of *Curvularia lunata* spores processed by flash freezing with liquid nitrogen (**a**) The surfaces of condiospore are little blurred as compared with native state preservation (Scale bar = 30 μm). (**b**) A semi-crytalline film formation on the outer surface of the spore (Scale bar = 20 μm). (**c**) Spore Dimension analysis of the spore (Scale bar = 20 μm). (**d**) Morphological characteristics of a single spore showing mild damage by slush nitrogen (Scale bar = 10 μm).

In sample flash frozen on liquid nitrogen, the fungal spores are clustered in crystalline aggregates. These spores were partly preserved and covered with crystalline film structures which are visible on top of the fungal spore. The spores septate and clavate were in the acceptable form. However, the outer surface layers of the spore were slightly leached. Interestingly, clear differences could be observed between frozen and those that were not. The condiospore were more loosely arranged and even isolated spore could be observed. The variation in spore dimensions were 8.84 μm long, 14.2 μm thick in the middle (the broadest part) and 3.04 μm inward groove at the apex (Fig. 4).

### Visualization of sample involving flash freezing and freeze drying

**Figure 5:**
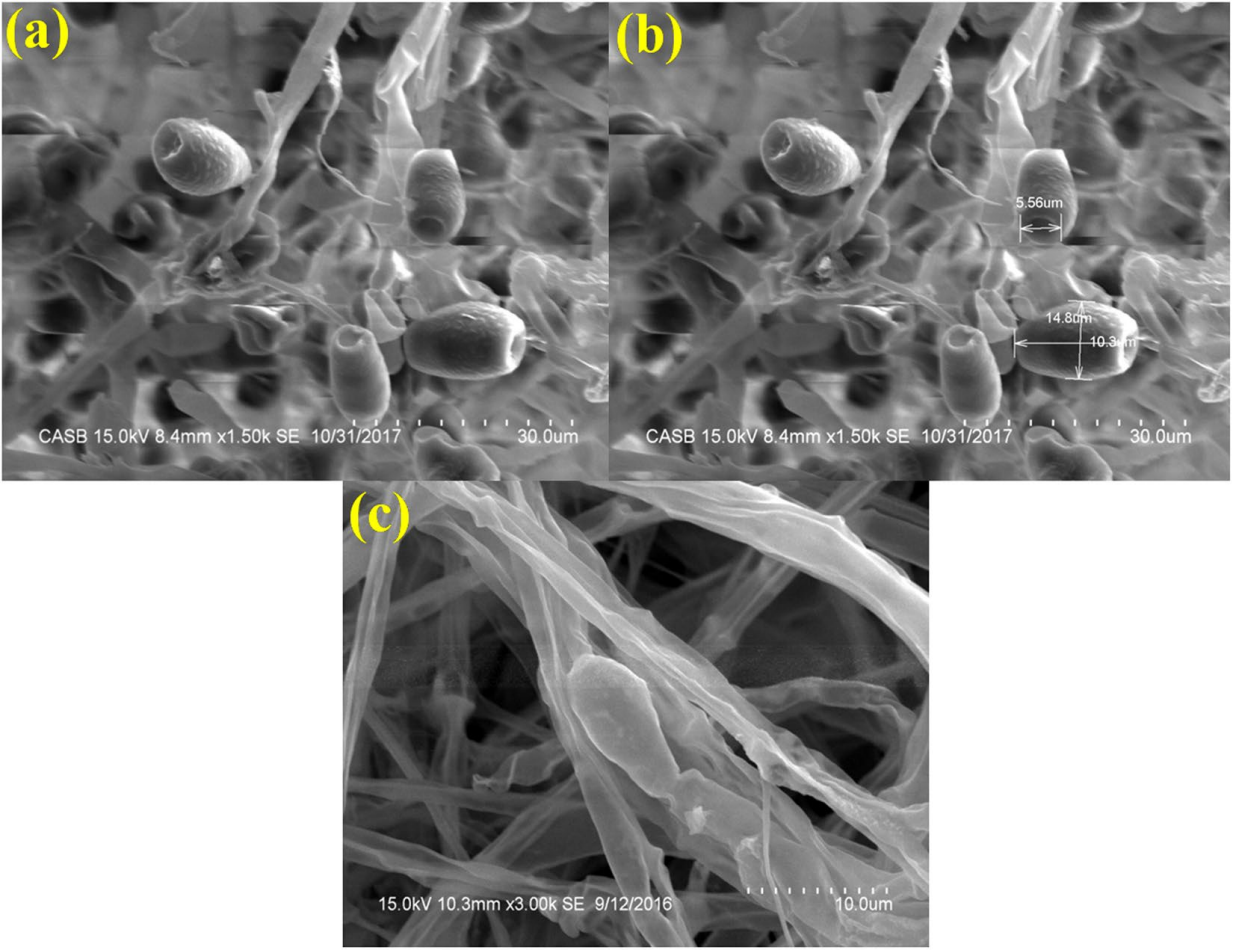
SEM analysis of *Curvularia lunata* spores processed by flash freezing with liquid nitrogen followed by freeze drying (**a**) Formation of crystal structures on the surface of conidiospore (Scale bar = 30 μm). (**b**) Spore dimension analysis (Scale bar = 20 μm). (**c**) Morphological characteristics of a single spore showing extensive damage on the structure (Scale bar = 10 μm).

In sample that were processed by Flash freezing followed by lyzophilization (freeze drying), the spore were found irregular in shape and shrivelled up with some shrinkage and few crystal deposits on the outer layer. The spores had rough texture and flask freezing has resulted in the formation of a dense layer of semi-pellucid ice crystals aggregates on top of the spores. The difference in spore dimension as compared with other methods employed includes 10.03 μm long, 14.8 μm thick in the middle (the broadest part) and 5.56 μm inward groove at the apex (Fig. 5).

### 3D Spores dimensional distribution

**Figure 6:**
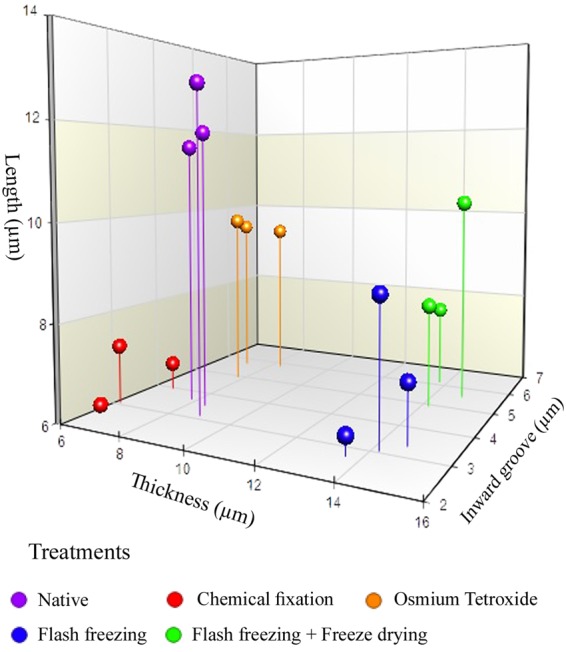
A 3D graph depicting the fungal spore dimensions imaged under SEM based on its length, thickness and inward groove in micometer scale (μm) when processed with various sample preparations treatments.[Violet dot-Native, Red dot- Chemical fixation, Orange dot- Post fixation with Osmium tetroxide (O_2_O_4_), Blue dot-Flash freezing and Green dot-Flash freezing followed by freeze drying].

The 3D graph revealed the difference in the length, thickness and inward groove of fungal spore dimensions in micrometer scale (μm) with respect to the five approaches used for the fungal sample preparation which were subsequently analysed under SEM imaging. The results conceded that among all the approaches used, native state succeeded in preserving the actual/natural spore dimension structures as compared with the other approaches used such as chemical fixation, post fixation, flash freezing and flash freezing followed by freeze drying respectively, where a drastic changes (in micrometer scale) in the spores dimension were observed which were exerted upon subjecting the fungal samples under treatment process. Though, the flash freezing and flash freezing followed by freeze dried partially preserved the spore dimension but it resulted in deposits of ice crystal on the spore surface resulting in reduced resolution. Among the treatments process used, chemical fixation and osmium tetroxide significantly resulted in extensive damage and spore dimension differences (Fig. 6).

## Discussion

Scanning Electron Microscope provides a precision three-dimensional visualization biological sample with high resolution and magnification and a common method employed to study the morphological characteristics and spatial relationships of biological samples. Generally, the samples in a SEM are visualized at ambient temperature with a high vacuum (ca.10^−8^ Torr), having the samples first been chemically fixed, dehydrated and then coated with a conductive material (e.g. gold) to prevent charge build-up from the electron beam. The specimen preparation for SEM at ambient temperature can be highly subtle^10–12^, but as it is not novel it will not be considered here, apart from noting that cautious specimen interpretation is essential in spite of preservation and imaging method^13^. To achieve a proper preservation and imaging of fungal sample, various techniques have been tried in our laboratory but with mixed success. Therefore, we were forced to develop a technique that preserves the native structure of the fungal spores of *Curvularia lunata* as model organism which belongs to phylum; ascomycetes causing the black kernel disease in rice (*Oryza sativa*). The procedures we developed in this study are simpler and just as fast without involving any equipment. In practice, the corresponding protocols may have to be adjusted and refined according to the nature of the samples. The developed method made it possible to look at the native spore structure of the fungus, but without the distortions introduced by chemical fixation and dehydration processes. Normally, visualization of fungal spores under SEM involves fixation with a fixative agent (glutaraldehyde or osmium tetroxide) for various amount of time and dehydration with graded ethanol series and dried by simple air-drying^14–17^. Simple air drying followed by chemical fixation resulted in substantial damage of the fungal sample. However, by growing the fungus on whatman No.1 filter paper and then simple air drying gave an acceptable and superior preservation of fungal spore structures and shape and size of the spores were comparatively better preserved than the other methods tested (Fig. 1).The samples treated with organic solvents like glutaraldehyde and osmium tetroxide have caused substantial alternation in the spore structure and heavy distortion in the outer layer which led to the deformation of the spores. Shrinkage on spore surface around the septum of the spore was also observed and probably it would have occurred during the process of dehydration rather than drying. This treatment totally collapsed the micro structure of the spores (Figs 2 and 3).Shrinking might also be a result of permutation of the specimens from one process to another treatment process. Since, a comparatively meagre surface distortion on the spores was attained by simple air drying, it is clear of the fact that the fixatives had a negative impact on the drying step leading to the extensive structural wreckage on the spores^18,19^. Thus, it is generally recommended that the fungal sample must be treated with glutaraldehyde or osmium tetroxide if sequential post fixation drying is to be attained by simple air drying. But, in spite of the presumption that fixing in glutaraldehyde and osmium teraoxide should invigorate the elasticity of cell walls by crossing-linking double bond configuration of the polymers and thus rendering them to resist cell collapse exerted during dehydrating forces. Proportionally, less fungal spore fractures were attained with simple air drying without fixation. So it was evident that the fixatives (glutaraldehyde and osmium tetroxide) negatively impacted in the drying step which caused a significant damages to the fungal spore^20–22^. The preliminary step involved in freeze-drying technique is to promptly freeze the sample so as to avoid frost-mist formation on the surface layer of the specimen.^23–25^.The promptness of freezing is probably the most pivotal factor on the final perpetuation of biological samples. Preferably, the samples can also be directly freeze-dried into liquid nitrogen (−196 °C), but as this leads in the formation of a gaseous/insulating layer (Leidenfrost effect) thus loses its contact with the sample^26,27^. In order to avoid the Leidenfrost effect, the samples can be flash frozen with liquid nitrogen and subjected to lyophilisation to remove the moisture. The freeze drying of the spores has resulted in freeze fractures and translocation of fungal spore and this technique provided preservation of fungal spore in some areas and surface had semi-crystalline aggregates^28^ (Fig. 5). Even though the quality of preservation was not far from native, but it was still comparable than that obtained from using chemical fixation such as glutaraldehyde and osmium tetroxide as fixative^29^ (Fig. 3). According to the theory, freezing the samples in liquid nitrogen slush should necessitate good freezing and compliant preservation of the samples^23,30^. However, in our experiments, adequate preservation of the fungal spore sample could not be achieved using this technique (Fig. 4).The introduction of structural artefacts of the spore at the surface by Au coating is yet another point worth considering^25^. From the 3D distribution graph of fungal spore dimension, the native state spore dimensions were used to compare the dimension difference with that of various treatments employed for sample preparation. Consequently, the chemical fixation by glutaraldehyde and osmium tetroxide had a major impact on the spore dimensions, where there was a drastic decrease in the length, thickness and inward groove in micrometer scale concluding the fact that the fixatives had a deleterious effects on spores as compared with native, flash frozen and flash freezing followed by freeze dried samples. Indeed, flash frozen and flash freezing followed by freeze dried relatively had a less impact on the fungal spore dimensions as compared with chemical and post fixation. However, was not comparably better than the native state which totally preserved the spore structures and its natural dimensions^31,32^ (Fig. 6**)**.

**Figure 7:**
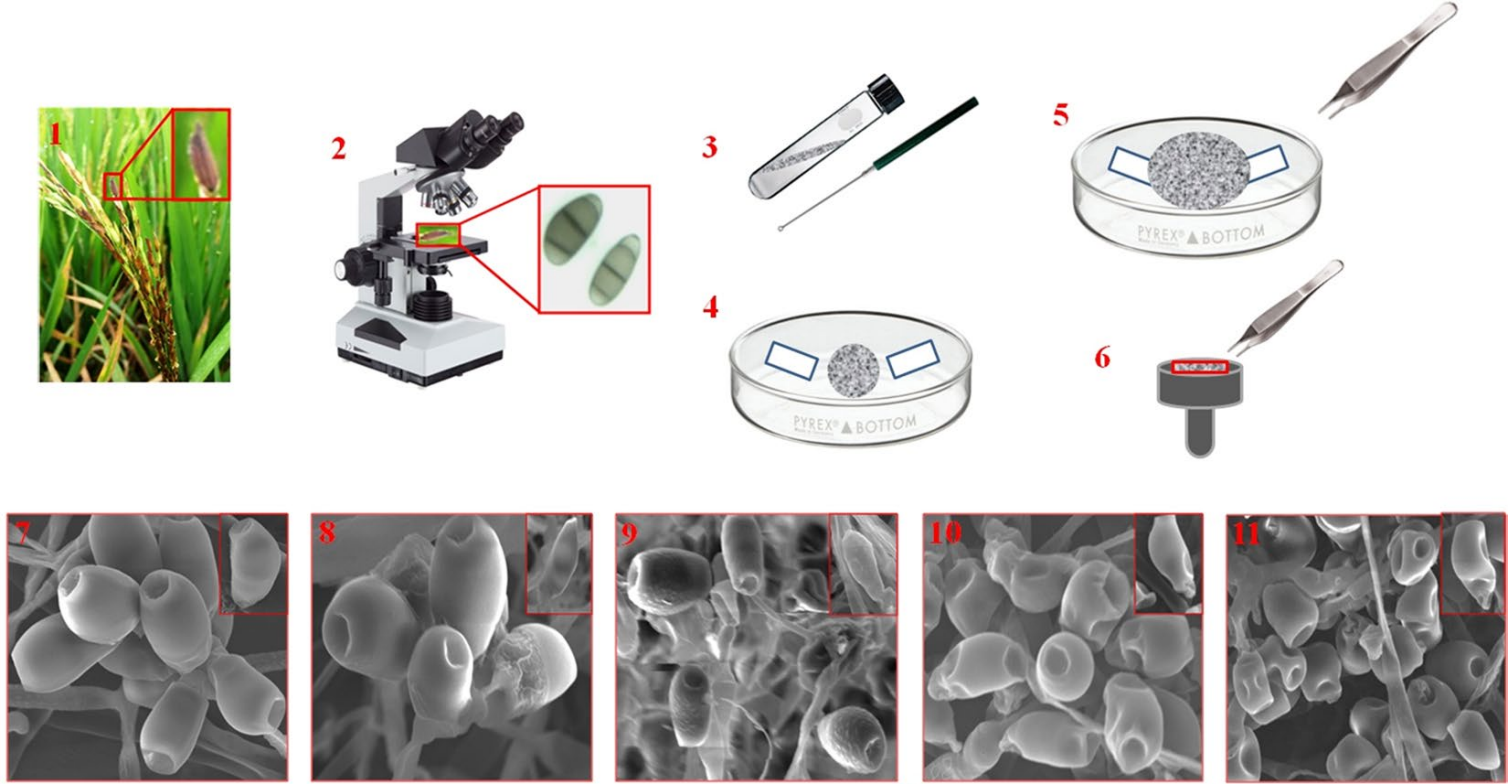
Schematic representation of sample preparation of *Curvularia lunata* spores for Scanning Electron Microscopy. (1) Isolation of *Curvularia lunata* from black kernel disease infected rice plants. (2) Morphological visualization of the fungal spores under light microscope (40x). (3) Maintenance of the fungal inoculums in PDA slant. (4) Inoculation and growth of the fungus on PDA medium overlaid on sterile Whatman No.1 filter paper strips, (5) Removal of the strips containing the fungal spores with sterile forceps. (6) The strips were placed on top of the stub for ion sputtering. (7) SEM images of the fungal spores at native state. (8) Flash freezing. (9) Flash freezing and freeze drying. (10) Chemical fixation with glutaraldehyde and (11) Chemical fixation with osmium tetroxide.

**Figure 8:**
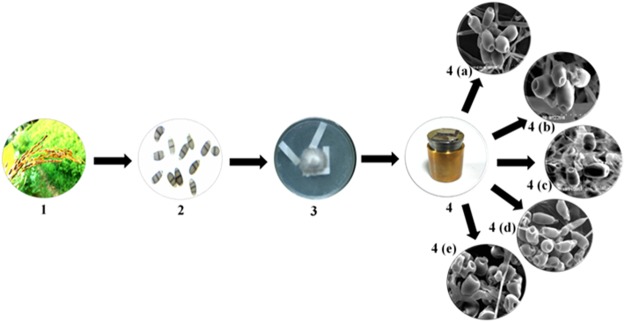
Overall design of fungal spore sample preparation for Scanning Electron Microscopy. Infected rice seedling caused by *Curvularia lunata* and its microscopic view (1, 2), Fungal grown on whatman No.1 filter paper (3), gold coating (4), The observed imaging includes; Native state (4a), flask freezing (4b), flask freezing and freeze dried (4c) glutaraldehyde (4d) and osmium tetroxide (4e).

From all the process performed, the imaging of fungal spores in native state gave possibly magnificent image as compared with other process used which required less technical equipment and is less time consuming^33^ (Fig. 1). Therefore, when planning an experiment which includes drying samples, the drying methods have to be carefully selected depending on the requirements of the underlying scientific question. Preferably, a harmonizing approach should be considered. Alternatively or complementarily, the sample could be analyzed in its frozen hydrated state using methods such as liquid nitrogen. This can provide an overview on the structure and composition of the sample and its relevant compartments and thus help us to decide on the preparation pathway^34^. As previously mentioned, it may not always be possible or suitable to use the method that is best suited for all fungal spore structural preservation. In these cases, this study can help to understand the results that were obtained using a certain way of preparation, and to reduce the risk of misinterpretation. In this study, we selected a complex type of fungal sample which belongs to ascomycetes family. Other fungal sample, may of course behave differently^20^. However, we believe that the issues described in this paper are relevant for a much larger variety of samples. This generally includes systems that consist of fungi used for taxonomical studies, especially in cases where description of fungal spores is essential. We believe that interpreted results compare constructively with published examples that take much more time to process. A schematic representation of the overall fungal sample preparation for SEM analysis has been illustrated in Figs 7 and 8. Finally, by comparing all the fungal sample preparation method used for the SEM imaging, fungal organism grown on upper surface of the whatman No.1 filter paper with thin layer of PDA served as the optimum and best sample preparation for SEM. The implication of the enhanced method developed affirms the underlying process for the preservation of fungal spore sample with an perspective to attain a targeted and focussed SEM images in their naturalistic and ‘true to life’ form.

## Conclusion

In this study, we have conclusively demonstrated how different preparation methods affect the structure and composition of fungal samples especially the spores. Advances in SEM methodologies have made it possible to explore a variety of questions in fungal biology. The ability to couple imaging with molecular genetic manipulations offers the possibility of deepening our understanding of how molecular mechanisms of the cell drive morphological and structural changes, to further elaborate fungal cytology. More broadly, the imaging with SEM will determine the molecular mechanisms alongside whole cell structural data, and the parallel development of technology has led to the integration of instrumentation for the simultaneous detection of different cellular properties and components (e.g. surface structure, rigidity, chemical composition, protein trafficking, etc.), enabling researchers to tackle complex biological problems in fungal biology. The establishment of new fungal sample preparation techniques opens the door to addressing many questions, which for fungi include unravelling the mechanisms that underscore host–pathogen interactions. The developed method also has the efficiency to be constructively used for studying fungal spore morphology which is an imperative requisite for fungal taxonomical studies. Furthermore, we expect that the enhanced methods will also have a role to play in developing new and better procedures for correlative light and electron microscopy.

## Methods

### Experimental methods

#### Model Organism

*Curvularia lunata*, a fungus causing the black kernel disease in rice (*Oryza Sativa*) and a model organism used in this study was isolated from the matured and diseased rice plants at the agricultural fields in Arakkonam, Tamil Nadu, India. The fungus was isolated from the infected rice kernels by inoculating onto the Potato Dextrose Agar (PDA) medium and incubating at 25 °C for 6 days. The conidia obtained from the fungus were used for SEM analysis using the preparation techniques prescribed hereunder.

#### Approaches for fungal sample preparation and visualization

The fungal organism was grown on a small piece of sterile Whatman No.1 filter paper strips (4 × 4 mm; L × B) containing Potato Dextrose Agar (PDA) medium. The PDA medium was poured into Petri dishes, the Whatman No.1 filter paper strips were placed on top of the semi solid PDA medium and allowed to solidify. After which, a thin layer of PDA medium was overlaid on top of the sterile filter strips and again allowed for solidification, which eventually resulted in the formation of a thin layer on the surface of the filter paper. Thereafter, the plate was inoculated with the fungal organism. The plates were incubated at 25 °C for 6 days until the fungus fully covers the strips. After the growth, the filter paper strips containing the fungus was carefully peeled off from the surface of the medium with the help of sterile forceps and used for different sample preparation processes.

#### Approach 1: Visualization of sample in its native state without any treatment

A small piece of Whatman No.1 paper (4 × 4 mm; L × B) containing the grown fungus was immediately placed on the carbon adhesive tape stuck on to the aluminium stub (26 mm) and coated with gold in real time for 100 seconds using an ion sputter (Hitachi, E-1010, Japan). The sputter coated sample was taken for imaging analysis using a Scanning Electron Microscope (Hitachi S-3400, Scanning Electron Microscope, Japan).

#### Approach 2: Visualization of sample after fixation

The Whatman strip containing the fungus was fixed with glutaraldehyde (2.5%, v/v) in phosphate buffer (10 mM; pH 7.4) for 4 h. The fixed samples were rinsed twice with deionized water and the samples were dehydrated with increasing concentrations of ethanol (10, 20, 40, 60, 80 and 100%) for 10 min each. After dehydration, the samples were dried at room temperature for 3 h. The dried samples were sputter coated and visualized as described above.

#### Approach 3: Visualization of sample involving fixation and post fixation

The fungal sample was first fixed with glutaraldehyde in phosphate buffer (10 mM; pH 7.4) for 30 min followed by washing with doubled distilled water four times and treated with osmium tetroxide (0.1% w/v) for 30 min. The samples were dried at room temperature for 3 h. The dried samples were sputter coated and visualized as described above.

#### Approach 4: Visualization of sample involving flash freezing

The flash-freezing setup consisted of a container filled with liquid nitrogen. A steel mesh (127.8 × 85.5 × 14.2 mm; L × W × H) was floated on top of the liquid nitrogen and the strip containing the fungus was placed on the steel mesh in such a way that the sample is not in direct contact with the liquid nitrogen and the fumes emanated from the liquid nitrogen freezes the sample. The frozen sample was immediately subjected to sputter coating and visualized with SEM as described above.

#### Approach 5: Visualization of sample involving flash freezing and freeze drying

The frozen sample described in the above step was freeze dried (Christ D-37520, Germany), sputter coated and visualized as described above.

#### Fungal spore dimension distribution graph

A three dimensional distribution graph for the fungal spore imaged under scanning electron microscope was plotted by using NCSS statistical analysis and graphical software program version 12.0.2^35^.

## Data Availability

The datasets generated and analyzed during the microscopy study are available from the corresponding author on reasonable request.
